# Lack of Evidence for Changing Virulence of HIV-1 in North America

**DOI:** 10.1371/journal.pone.0001525

**Published:** 2008-02-06

**Authors:** Joshua T. Herbeck, Geoffrey S. Gottlieb, Xiuhong Li, Zheng Hu, Roger Detels, John Phair, Charles Rinaldo, Lisa P. Jacobson, Joseph B. Margolick, James I. Mullins

**Affiliations:** 1 University of Washington School of Medicine, Seattle, Washington, United States of America; 2 Johns Hopkins University, Bloomberg School of Public Health, Baltimore, Maryland, United States of America; 3 University of California Los Angeles School of Public Health, Los Angeles, California, United States of America; 4 Northwestern University Medical School, Chicago, Illinois, United States of America; 5 University of Pittsburgh Graduate School of Public Health, Pittsburgh, Pennsylvania, United States of America; National AIDS Research Institute, India

## Abstract

**Background:**

Several long-term cohort studies and *in-vitro* fitness assays have resulted in inconsistent reports on changes in HIV-1 virulence, including reports of decreasing, stable, and increasing virulence over the course of the AIDS pandemic. We tested the hypothesis of changing HIV-1 virulence by examining trends in prognostic clinical markers of disease progression from 1984 to 2005 among nearly 400 antiretroviral-naïve participants in the United States Multicenter AIDS Cohort Study (MACS), a longitudinal study of HIV infection in men who have sex with men (MSM).

**Methodology/Principal Findings:**

Because clinical AIDS endpoints could not be used (due to antiretroviral therapies and prophylaxis), three prognostic markers of disease progression were used as proxies for HIV-1 virulence: plasma viral RNA load and CD4+ T cell count at “set point” (between ∼9 and ∼15 months after seroconversion), and rate of CD4 cell decline within three years after seroconversion. We performed multivariate analyses of the association between these markers and seroconversion year, with covariates including MACS site, race/ethnic group, seroconversion age, and CCR5Δ32 status. No statistically significant association was found between year of seroconversion and “set point” plasma viral load (at ∼9 months after seroconversion: slope = −0.004 log_10_ copies/mL/year, p = 0.76; at ∼15 months: slope = −0.005 log_10_ copies/mL/year, p = 0.71), CD4 cell count after seroconversion (at ∼9 months: slope = −0.112 cells/µL/year, p = 0.22; at ∼15 months: slope = −0.047 cells/µL/year, p = 0.64), or rate of CD4 cell decline over the first three years after seroconversion (slope = −0.010 cells/ul/yr^2^, p = 0.88).

**Conclusions/Significance:**

The lack of significant trends from 1984 to 2005 in these prognostic markers of HIV disease progression suggests no major change in HIV-1 virulence over the AIDS pandemic in MSM in the US.

## Introduction

Whether HIV-1 has become more or less virulent since its introduction into the human population has significant implications for the course of the AIDS pandemic. Over the last two decades, several studies of long-term cohorts have resulted in conflicting data on this question, including reports of increasing [1]–[4], stable [5]–[10], and decreasing virulence [11]. There are also contrary reports of changing HIV-1 virulence based on *in-vitro* competitive viral fitness data used as proxies for virulence. Arien *et al*. [12], [13] found HIV-1 replication capacity was greater in early (1986–1989) than in recent isolates (2002–2003) from Belgium (n = 24), and suggested that HIV may be attenuating over time. In contrast, Gali *et al.* [14], using the same *in-vitro* methods in an Amsterdam cohort (n = 25), found that HIV replication capacity was greater in recent (late 1990s) than in earlier isolates (mid-1980s), suggesting the opposite conclusion. In a recent large study of 22 cohorts from Australia, Canada, and Europe, Dorrucci *et al*. (2007) found trends of decreasing CD4+ T-cell counts (n = 3687) and increasing viral loads (n = 1584) in measurements at ∼4 months after seroconversion between 1985 and 2002, suggestive of increasing HIV-1 virulence over time [1]–[4].

Studies of host-pathogen interactions suggest that hosts and pathogens can co-adapt to each other, with pathogens becoming less virulent over time [15]. Possible examples of this phenomenon are primate lentiviruses such as SIVcpz (HIV-1) in chimpanzees and SIVsm (HIV-2) in sooty mangabeys, viruses that cause no apparent disease in their natural hosts [16], [17]. Attenuation of HIV-1 could result from the decrease in viral fitness that often accompanies viral evolution away from host cytotoxic T-lymphocyte (CTL) and humoral immune responses [18], [19]. In addition, data suggest that the accumulation of drug resistance mutations can reduce the replication capacity of HIV-1 [20], which could concomitantly lead to reduced virulence. Conversely, it is also possible that HIV-1 may be under selection for increased virulence in the human population, as higher viral loads are associated with increased probability of transmission [21]–[23].

Pathogen virulence is most commonly measured in host mortality [15]. For HIV-1, the most direct measure of virulence is the time from initial infection to the development of clinical AIDS and death. Thus, evidence for decreased virulence of HIV-1 would be a lengthening interval from infection to AIDS and death over the course of the epidemic. We tested the hypothesis of changing HIV-1 virulence by examining trends in rate of progression of HIV-1 infection in men followed in the Multicenter AIDS Cohort Study (MACS) from 1984 to 2005. Prognostic markers of disease progression had to be used as endpoints, because direct measures of this rate, such as time from seroconversion to AIDS and death, have been dramatically affected over the second half of this interval by the advent of potent antiretroviral therapies and by progress in prophylaxis and treatment of opportunistic infections and AIDS-related malignancies. Of the multiple prognostic markers of HIV-1 disease progression, we selected 1) the viral set point, defined as the plasma viral RNA level at the second or third semiannual visit after seroconversion, because it has been shown to be the strongest single early predictor of disease course [24]–[27], and 2) the CD4 cell count and the slope of its decline, because these markers have commonly been used in cohort-based analyses of long-term changes in HIV virulence [1], [3], [6], [7].

## Results

The demographic characteristics of the study population are shown in Table 1. The median plasma viral load at the second seropositive visit (∼9 months after seroconversion) was 4.485 log_10_ copies/mL (n = 357); at the third seropositive visit (∼15 months) this figure was 4.399 log_10_ copies/mL (n = 318) (Table 1). The median CD4 cell count was 624 cells/µL (n = 375) at the second visit and 588 cells/µL (n = 348) at the third visit (Table 1).

**Table 1:** Summary of participants and data.

Age at seroconversion		N	%
	<30	123	29.78
	30–39.9	194	46.97
	40–49.9	71	17.19
	> = 50	25 (overall)	6.05 (Median (IQR))
		413	33.58 (29.2, 39.3)
Race		N	%
	Black	30	7.26
	Hispanic	23	5.57
	Other	1	0.24
	White	359	86.92
Center		N	%
	Baltimore	105	25.42
	Chicago	80	19.37
	Los Angeles	130	31.48
	Pittsburgh	98	23.73
CCR5Δ32 heterozygous		N	%
	-	337	83.21
	+	68	16.79
CD4+ T-cell count		N	Median (IQR)
	1st	408	730 (539, 921)
	2nd	375	624 (454, 837)
	3rd	348	588 (453, 799)
	2nd/3rd mean	409	600 (459, 817)
Plasma log10 viral load		N	Median (IQR)
	1st	375	4.434 (3.90, 4.96)
	2nd	357	4.485 (3.95, 4.87)
	3rd	318	4.399 (3.95, 4.77)
	2nd/3rd mean	384	4.499 (3.99, 4.81)
Years		viral load	CD4+ T-cell count
		N	N
	1984–1989	303	303
	1990–1994	63	88
	1995–1999	7	7
	2000–2005	11	11

Demographics of participants examined in this study; Characteristics of prognostic markers of HIV-1 disease at the first three visits (1^st^, 2^nd^, 3^rd^) or a combination of the data available from the 2^nd^ and 3^rd^ visit after seroconversion (∼3, ∼9, and ∼15 months) in MACS participants from 1984 to 2005, prior to any antiretroviral therapy; Number of participants with prognostic marker data by year in quartiles, using the mean of the second (∼9 mos.) and third (∼15 mos.) seropositive visits, or one of these visits if data was not available from both.

We found no significant trends in set point plasma viral load over the 20-year study period, in either univariate or multivariate models. For the viral load at the second seropositive visit, the slope was −0.004 log_10_ copies/mL/year, (p = 0.76; Table 2); for the third seropositive visit the slope was −0.005 log_10_ copies/mL/year (p = 0.71; Table 2). Among the covariates examined in the multivariate model, only heterozygosity of the coreceptor allele CCR5Δ32 showed a significant association with viral setpoint (Table 2) (no participant was CCR5Δ32 homozygous).

**Table 2:** Multivariate analyses of prognostic markers of HIV-1 disease.

	Plasma viral load (log10)			CD4 cell count*		
	copies/mL/year	cells/mL/year		
a)	N = 357			N = 375		
	Estimate	*P*	95% CI	Estimate	*P*	95% CI
Calendar year of SC	−0.004	0.76	−0.030, 0.021	−0.112	0.22	−0.289, 0.066
Age at SC (per 10 yr increase, centered at 30 yrs)	0.088	0.11	−0.022, 0.199	−0.267	0.5	−1.036, 0.503
MACS site (Pittsburgh used as reference)						
Baltimore	−0.091	0.47	−0.337, 0.156	1.411	0.1	−0.293, 3.114
Chicago	−0.107	0.42	−0.369, 0.154	−0.388	0.67	−2.202, 1.426
Los Angeles	−0.129	0.28	−0.364, 0.106	0.575	0.48	−1.004, 2.153
Race/ethnicity (White, non-Hispanic used as reference)						
Black, non-Hispanic	0.098	0.55	−0.228, 0.423	0.142	0.91	−2.254, 2.537
Hispanic	−0.12	0.5	−0.471, 0.230	−0.269	0.84	−2.855, 2.318
Other	−0.265	0.73	−1.778, 1.248	−6.062	0.28	−17.041, 4.916
CCR5Δ32						
Heterozygous	−0.441	<0.0001	−0.655,−0.227	0.672	0.4	−0.880, 2.223
b)	N = 318			N = 348		
	Estimate	*P*	95% CI	Estimate	*P*	95% CI
Calendar year of SC	−0.005	0.71	−0.032, 0.022	−0.047	0.64	−0.244, 0.151
Age at SC	0.038	0.49	−0.068, 0.144	0.024	0.95	−0.758, 0.805
MACS site						
Baltimore	−0.101	0.41	−0.338, 0.137	1.684	0.06	−0.069, 3.437
Chicago	0.01	0.92	−0.237, 0.258	−1.288	0.17	−3.128, 0.548
Los Angeles	−0.038	0.74	−0.263, 0.188	−0.088	0.92	−1.732, 1.556
Race/ethnicity						
Black, non-Hispanic	−0.041	0.8	−0.359, 0.277	−0.03	0.98	−2.610, 2.550
Hispanic	0.002	0.99	−0.327, 0.331	−0.018	0.99	−2.637, 2.601
Other	−0.318	0.65	−1.673, 1.037	−4.28	0.44	−15.121, 6.561
CCR5Δ32						
Heterozygous	−0.441	<0.001	−0.641, −0.241	0.297	0.7	−1.242, 1.836

SC = seroconversion, CI = confidence interval, * square root-transformed

a) Data from second seropositive visit (∼9 months after estimated date of SC) versus calendar year of SC, 1984 to 2005. b) Data from third seropositive visit (∼15 months after estimated date of SC) versus calendar year of SC, 1984 to 2005.

In the CD4 cell count analysis, we found no significant association between calendar year of seroconversion and the square root-transformed CD4 cell count at either the second or third seropositive visits, in either univariate or multivariate analysis. Specifically, the slope of the square root-transformed CD4 cell count at the second seropositive visit was −0.112 cells/µL/year (p = 0.22; Table 2); and at the third seropositive visit this slope was −0.047 cells/µL/year (p = 0.64; Table 2). Similarly, there was no correlation between calendar year of seroconversion and rate of CD4 cell decline over the first three years after seroconversion, in either univariate or multivariate models (slope = −0.010 cells/ul/yr^2^, p = 0.88; Table 3). None of the covariates examined was significantly associated with either the CD4 cell count at the second or third seropositive visit, or its slope over the first three years after seroconversion (Table 2, Table 3).

**Table 3:** Multivariate analyses of prognostic markers of HIV-1 disease.

a)	Plasma viral load (log10)			CD4 cell count*		
	copies/mL/year			cells/mL/year		
	N = 384			N = 409		
	Estimate	*P*	95% CI	Estimate	*P*	95% CI
Calendar year of SC	−0.005	0.66	−0.030, 0.019	−0.138	0.1	−0.302, 0.025
Age at SC	0.092	0.07	−0.009, 0.193	−0.094	0.79	−0.791, 0.604
MACS site						
Baltimore	−0.15	0.19	−0.372, 0.072	2.083	0.01	−0.529, 3.636
Chicago	−0.078	0.51	−0.311, 0.156	−0.407	0.62	−2.037, 1.223
Los Angeles	−0.059	0.58	−0.270, 0.151	0.541	0.46	−0.891, 1.973
Race/ethnicity						
Black, non-Hispanic	0.083	0.57	−0.206, 0.372	−0.403	0.71	−2.539, 1.732
Hispanic	−0.064	0.69	−0.378, 0.250	−0.529	0.65	−2.816, 1.759
Other	−0.311	0.67	−1.731, 1.108	−5.244	0.32	−15.611, 5.123
CCR5Δ32						
Heterozygous	−0.426	<0.001	−0.622,−0.231	0.644	0.37	−0.764, 2.053
b)	Rate of CD4 cell decline†					
	cells/uL/yr2					
	N = 422					
	Estimate	*P*	95% CI			
Calendar year of SC	−0.01	0.88	−0.133, 0.114			
Age at SC	0.418	0.12	−0.946, 0.111			
MACS site						
Baltimore	0.995	0.09	−0.166, 2.157			
Chicago	0.323	0.61	−0.902, 1.548			
Los Angeles	0.822	0.13	−0.243, 1.888			
Race/ethnicity						
Black, non-Hispanic	−1.613	0.05	−3.211, −0.015			
Hispanic	−2.051	0.02	−3.756, −0.347			
Other	0.702	0.86	−7.197, 8.601			
CCR5Δ32						
Heterozygous	0.739	0.17	−0.326, 1.803			

SC = seroconversion, CI = confidence interval, † within 3 years of SC, * square root-transformed

a) Data using the mean of the second (∼9 months) and third (∼15 months) seropositive visits, or one of these visits if data was not available from both. b) The slope of CD4 cell decline within the first three years after SC.

**Figure 1:**
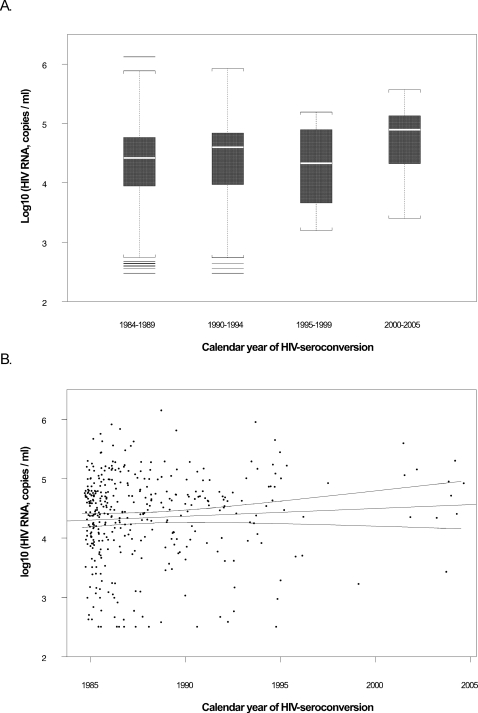
RNA viral loads through time. A) Boxplot for the mean of log10 HIV RNA at second and third seropositive visits (∼9 and ∼15 months after estimated date of seroconversion) by calendar period of seroconversion. The horizontal line in the interior of the box denotes the median; and the height of the box is equal to the interquartile distance (IQD). The whiskers (the dotted lines extending from the top and bottom of the box) extend to the extreme values of the data or a distance 1.5×IQD from the center, whichever is less. The horizontal lines falling outside the whiskers represent outliers. B) Relationship between the mean of log10 HIV RNA at second and third seropositive visits and calendar year of seroconversion. Three solid lines are the fitted regression line and its 95% confidence bands from univariate linear regression (note that slope estimates reported in manuscript text are from multivariate analysis).

**Figure 2:**
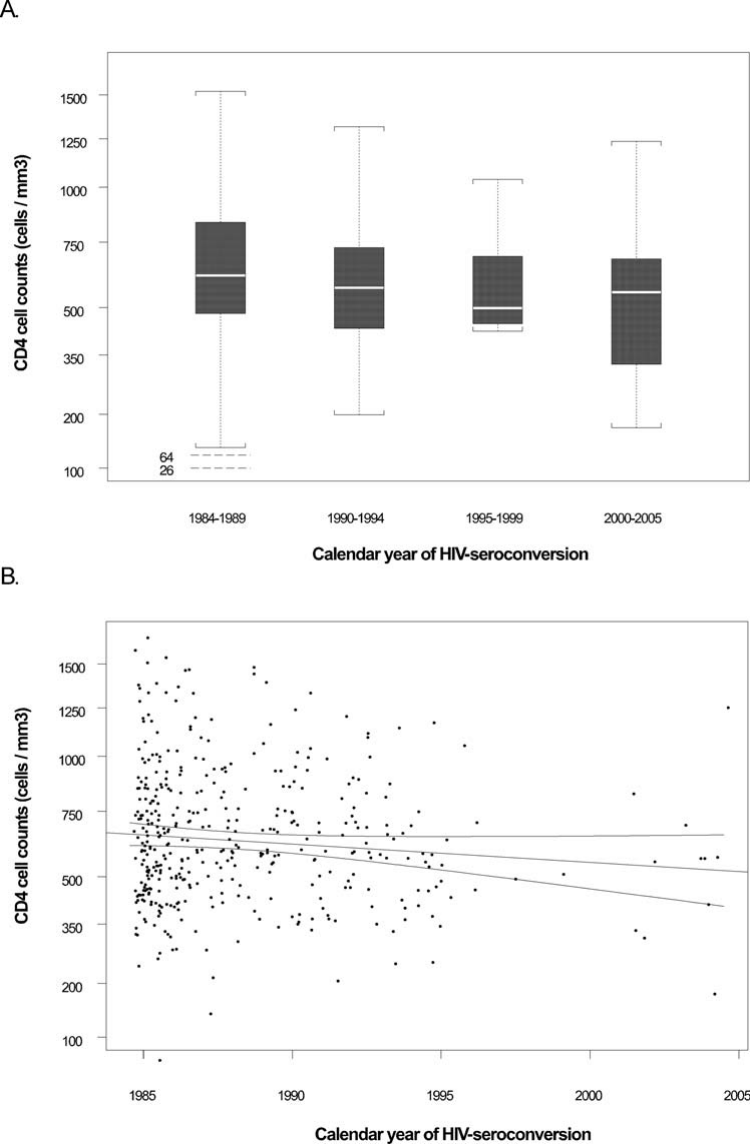
CD4+ T cell levels through time. A) Boxplot for the mean of square-root transformed CD4+ T-cell count at second and third seropositive visits (∼9 and ∼15 months after estimated date of seroconversion) by calendar period of seroconversion. Boxes are defined as in the legend to Figure 1. B) Relationship between the mean of square-root transformed CD4+ T-cell count at second and third seropositive visits and calendar year of seroconversion. Three solid lines are the fitted regression line and its 95% confidence bands from univariate linear regression (note that slope estimates reported in manuscript text are from multivariate analysis).

In the analysis of the mean of the second and third seropositive visits (if data was available for both visits, only one visit if only visit had data available), we found no significant temporal trend in viral load (n = 384, slope = −0.005 log_10_ copies/mL/year, p = 0.66) (Table 3) (Figure 1). We found a slight decreasing trend in square-root transformed CD4 cell count (n = 409, slope = −0.138 cells/µL/year, p = 0.10) (Table 3) (Figure 2). These trends translate to decreases of of ∼0.1 log_10_ copies/mL in viral load set point and ∼7 cells for the CD4 cell count over the 20 year study period; neither of these values is clinically significant.

All of the above analyses were also done using calendar years in bins comprised of data from 5 year intervals, and yielded results consistent with the above analyses of individual calendar years. We also tested for a trend over time in the lag between estimated date of seroconversion and the second and third seropositive visits; no trend was found.

## Discussion

We tested for associations between calendar year of seroconversion and three prognostic markers of disease progression in the MACS cohort between 1984 and 2005. Our results showed no significant trends in set point plasma viral RNA load, CD4 cell count after seroconversion, or the rate of CD4 cell decline in the first three years after seroconversion. Moreover, estimates of change in these markers over time were very close to zero. Thus, the results of this study do not support the hypothesis that there has been any important change in the virulence of HIV-1 over this time period in this cohort. This is consistent with recent findings that revealed no long-term changes in virulence over 20 years in a Swiss cohort [10], and with a previous analysis of MACS participants that reported stable rates of CD4 cell loss between 1984 and 1991 [6].

Our results are contrary to those from studies of long-term cohorts have found decreasing [11] or increasing [1]–[4] virulence over time. The largest study to date, by Dorrucci *et al*. [4], analyzed Australian, Canadian, and European cohorts from 1985 to 2002, and found a decreasing trend in the first CD4 cell count (n = 3687) and an increasing trend in the first viral load (n = 1584) after seroconversion. That study used the first CD4 cell count after seroconversion, with a median lag time between seroconversion and CD4 cell count of ∼4 months; we used CD4 cell counts at ∼9 and ∼15 months after seroconversion. In order to more directly compare our and Dorrucci *et al.*'s results, we estimated trends from 1984 to 2005 in the plasma viral load and CD4 cell count at the first seropositive visit in the MACS cohort, corresponding to ∼3 months after seroconversion. We again found no significant association between viral load and seroconversion year (n = 375, slope = −0.011 log_10_ copies/mL/year, p = 0.48, 95% CI –0.042 to 0.020), but did observe a decreasing trend in square-root transformed CD4 cell count (n = 408, slope = −0.194 cells/µL/year, p = 0.03, 95% CI –0.363 to –0.024). We suspect this result, and the discrepancy between our analyses at ∼9 and ∼15 months after seroconversion and those of Dorrucci *et al*., may be due to the significant CD4 cell fluctuations that occur during acute and early infections [25], [28]. By ∼9 and ∼15 months after seroconversion CD4 cell counts have likely recovered and stabilized from these fluctuations. It will be beneficial to examine trends in viral load and CD4 cell count at later timepoints after seroconversion in the Dorrucci *et al.* cohorts.

Our results are also contrary to the recent inference of HIV-1 attenuation based on *in-vitro* competitive fitness data between early (1986–1989) and recent isolates (2002–2003) from Belgium [12], [13]. However, Gali *et al*. [14] used the same fitness assay approach in a study comparing Amsterdam Cohort Studies samples from the mid-1980s against samples from the late 1990s, matched for time since seroconversion, coreceptor usage, and viral subtype. They found that the recent isolates had higher fitness than earlier isolates, and suggested HIV-1 virulence may be increasing in this cohort. The number of isolates tested in these two studies was small (n = 24 and 25, respectively). As hinted at by these discrepant results, it is possible that changes in *in-vitro* fitness are not directly or easily compared to clinical prognostic markers or virulence in humans, and that larger sample sizes may be required in these assays.

Host factors (and potentially other unknown factors) can affect rates of disease progression independently of viral load [29]; it is possible that existing variation in progression rates to AIDS or death in the MACS is not reflected in the viral load or CD4 cell count data we examined. However, it is unlikely that allele frequencies of particular host factors (*e.g.* CCR5Δ32, CCL3L1) have changed significantly in humans between 1984–2005. HIV-1 subtypes can vary in disease progression rates independently from viral load as well [30]. Yet we believe that all, or at least the vast majority, of the subjects in this study are infected with subtype B, as there have been no reports of non-subtype B virus in the MACS to this date.

Our study has some limitations. First, seroconverters in the MACS cohort were significantly more frequent in the first decade of the study period than in later years. Second, even though the MACS followed large numbers of seroconverters until clinical AIDS or death, we were unable to use those disease outcomes in our analyses because calendar year is linked to progress in HIV/AIDS disease management (*e.g*., prophylaxis and treatment of opportunistic infections, and development of more potent antiretroviral therapies). Thus, we had to rely on prognostic markers to study trends in HIV-1 disease progression rates. Third, because we excluded men who started antiretroviral therapy, there is a possible selection bias; men who experienced disease progression would not contribute to our analysis, and any decrease of the mean time from seroconversion to first therapy (into the second or third seropositive visit) over the study period would not be detected. However, the number of men excluded is small (for viral load analysis, n = 5 at first seropositive visit, n = 14 at second, n = 16 at third; for CD4 analysis, n = 6 at first seropositive visit, n = 20 at second, n = 18 at third; for CD4 slope analysis, n = 28 in the first three years after seroconversion), and this likely did not impact our analyses. Fourth, we could not correct for the changes in laboratory methods for CD4 or viral load measurements over this time period, as the these changes correlate completely with year (and we are looking for changes in estimates of these markers by year). This issue is one that plagues all similar studies, including Dorrucci *et al.* (2007). Lastly, although we found no significant changes in set point viral load over the 20 years of observation, the 95% confidence intervals encompass a range of ∼−0.6 to +0.4 log_10_ copies/mL over this period (Table 2), which is greater than the 0.3 log_10_ copies/mL cut-off that has been used as a “clinically significant” change in this marker [24], [25].

In conclusion, our data suggest that there has been no change in HIV-1 virulence over the last 20 years in the MACS cohort. However, it is unclear if more frequent and earlier use of highly active antiretroviral therapy will have an eventual negative impact on transmission and subsequent HIV virulence, or if the association between higher viral loads and transmission risk will eventually select for greater virulence in the human population.

## Materials and Methods

### Study population

The Multicenter AIDS Cohort Study (MACS) is an ongoing prospective study of the natural and treated histories of HIV-1 infection in homosexual and bisexual men conducted by sites located in Baltimore, Chicago, Pittsburgh and Los Angeles [31]. A total of 6,972 men have been enrolled during three enrollment periods: April 1984 through March 1985 (n = 4954), April 1987 through September 1991 (N = 668), and October 2001 and August 2003 (n = 1350). MACS participants were seen during semi-annual visits at one of the study sites where they underwent standardized questionnaires, physical examinations and laboratory assessments [31]. Study participants in the MACS provided written informed consent and IRB approval for this study was obtained from the University of Washington and the MACS parent institutions.

At the time of the present analysis, there were 2884 men who had entered the study as HIV-seroprevalent, and 596 men with a known date of seroconversion. Estimated date of seroconversion was the median time between the last seronegative visit and the first seropositive visit. For each participant, we included only data from visits prior to the start of any antiretroviral treatment (including any mono-, dual-, or highly active antiretroviral therapy (ART)). We further restricted the analysis to those 483 seroconverters who had less than one year between their last seronegative and first seropositive visits. Of these, 357 had plasma viral load measurements at ∼9 months after estimated date of seroconversion, and 318 had viral load measurements at ∼15 months after seroconversion date. The corresponding numbers for CD4 cell counts were 375 and 348, respectively (Table 1). To increase the sample sizes in our analyses, we also tested for associations between prognostic markers and year of seroconversion using combined data from the second and third seropositive visits. The mean of the two visits was used if data was available for both visits; if data from only one of these visits was available, that value was used. This approach increased overall numbers included to 384 for plasma viral load and 409 for CD4 cell count, and also reduced potential lag-time or outlier bias.

The 596 men with known dates of seroconversion included 59 men from Pittsburgh who enrolled in the MACS already seroprevalent but with a known seroconversion date; 49 of these men had less than one year between their last seronegative and first seropositive visits, and 11 of these had viral load and CD4 measurements within 6 to 18 months of seroconversion. For our tests of seroconversion year versus rate (slope) of CD4 cell decline, 412 men contributed at least two measurements within three years following seroconversion and had no missing information on race, age, and CCR5Δ32 genotype, and thus contributed to the multivariate analysis.

### Laboratory Methods

At each semiannual MACS visit, absolute CD4+ T lymphocyte counts were obtained by flow cytometry [32] and an automated complete blood count and differential determined. HIV-1 RNA was measured in stored plasma samples by reverse-transcription polymerase chain reaction (RT-PCR) (AMPLICOR HIV-1 MONITOR Test, Roche Diagnostics, Nutley, NJ) with assay detection limits of 400 (Standard Assay) and 50 (UltraSensitive Assay) copies/mL.

### Statistical Analysis

We performed univariate and multivariate linear regression of the association between date of seroconversion (independent) and prognostic markers of HIV infection (dependent): 1) plasma viral load (log_10_ copies/mL) at the second (∼9 months) and third (∼15 months) seropositive visits; 2) the CD4 cell count (square root-transformed to achieve approximate normality) at the second and third seropositive visits; and 3) slope of the CD4 cell decline per year within three years after seroconversion. Covariates included MACS center location (Baltimore, Chicago, Los Angeles, Pittsburgh), race (Black non-Hispanic, Hispanic, White non-Hispanic, and other), age at seroconversion (centered at 30 years old), and CCR5 gene status (homozygous wild type vs. heterozygous Δ32). Statistical associations were performed using SAS 9.1 (SAS institute, Cary NC). Graphs for figures were generated using S-Plus 6.2 (Insightful Corporation, Seattle, WA).
